# Pine-Extracted Volatile Oils Suppress Root Rot in *Psammosilene tunicoides* Through Direct Antifungal Activity and Rhizosphere Microbiome Modulation

**DOI:** 10.3390/plants15142228

**Published:** 2026-07-21

**Authors:** Li-Juan Wang, Fei Ji, Shu-Yun Qi, Qiao-Feng Li, Min Zhao, Chun-Ju Xu, Ying-Tao Li, Ai-Li Zhang

**Affiliations:** 1 Yunnan Key Laboratory of Chinese Medicine Processing, Yunnan University of Chinese Medicine, Kunming 650500, China; wanglijuan@ynucm.edu.cn (L.-J.W.); jifei@yuncm.edu.cn (F.J.); qishuyun@ynucm.edu.cn (S.-Y.Q.); liqiaofeng@ynucm.edu.cn (Q.-F.L.); zhaomin8@ynucm.edu.cn (M.Z.); xuchunju@ynucm.edu.cn (C.-J.X.); 2 School of Chinese, Yunnan University, Kunming 650500, China

**Keywords:** *P. tunicoides*, root rot, pine-derived volatile oils, antifungal activity, rhizosphere microbiome

## Abstract

Frequent outbreaks of root rot in *Psammosilene tunicoides* W. C. Wu & C. Y. Wu severely compromise the quality of its medicinal materials and hinder its large-scale cultivation. Interestingly, wild *P. tunicoides* growing under pine trees rarely experience this disease. To explore the potential basis of root rot suppression, we evaluated the direct antifungal activity of pine-derived volatile oils and the associated changes in the rhizosphere microbiome. GC-MS showed that pine turpentine was dominated by α-pinene (45.50%) and longifolene (28.20%). In vitro assays confirmed its highly efficient inhibition (81.65–94.71%) against major root rot pathogens in *P. tunicoides*. Beyond direct antifungal effects, metagenomic analysis indicated that volatile oil (SYR) treatment was associated with shifts in the rhizosphere microbiome, including increased relative abundances of potentially beneficial taxa, such as *Paenibacillus*, *Trichoderma*, and *Geosiphon*. Pine volatiles might be associated with shifts in the rhizosphere microbial community of *P. tunicoides*, potentially involving plant-mediated changes in root exudation and the enrichment of certain beneficial microbes. However, it remains to be further elucidated regarding the specific mechanisms underlying these community changes. Functional prediction of the microbial community suggested a predominance of metabolic pathways, secondary metabolite biosynthesis, and flagellar assembly in the SYR group. Conclusively, pine volatiles may contribute to root rot suppression through two potential processes: direct pathogen inhibition and beneficial microbiome enrichment. This study provides a theoretical basis for establishing sustainable agroforestry co-planting systems for *P. tunicoides*.

## 1. Introduction

*Psammosilene tunicoides* W. C. Wu & C. Y. Wu (*P. tunicoides)*, an endemic medicinal species in southwestern China, has long been valued in ethnomedicine for its pronounced anti-inflammatory, antioxidant, and immunomodulatory properties. With an extensive application in traditional Chinese medicine, *P. tunicoides* serves as a key ingredient in well-known formulations such as “Yunnan Baiyao” [1,2]. However, its wild populations face a sharp decline owing to enormous commercial value and persistent overharvesting. At present, the species has been designated as a National Class II Key Protected Wild Plant (as per the 2021 “List of National Key Protected Wild Plants”) and was assessed as “Endangered” in the 2013 “China Biodiversity Red List: Volume of Higher Plants”. To date, commercial demand for this medicinal material has been largely met through cultivation. Nevertheless, root rot during its cultivation has emerged as a major constraint that leads to significant reduction in both yield and quality, thereby impeding the sustainable development of the industry [3]. Indeed, chemical pesticides can provide short-term control, which, however, may often induce environmental contamination, residue accumulation, and pathogen resistance [4]. This highlights an urgent need for identifying disease control strategies that are both effective and environmentally friendly.

In our preliminary work, field investigations revealed that wild populations of *P. tunicoides* are usually grown beneath *Pinus yunnanensis* Franch. forests, where the incidence of root rot was markedly lower [5]. Forest understory ecosystems exhibit high biodiversity, within which plants interact through the release of secondary metabolites (e.g., phenolics, terpenoids, and alkaloids), forming a distinctive “chemical language” that mediates complex ecological relationships [6,7,8]. Existing data have documented that root exudates and decomposition products of plant litter can directly suppress soil-borne pathogens [9]. These compounds may also influence the metabolic activities of neighboring plants and may be associated with changes in rhizosphere microbiota, which may enhance host resistance to pathogens [10]. Alternatively, such chemical cues may trigger systemic acquired resistance (SAR) or induced systemic resistance (ISR) in adjacent plants, further strengthening their defensive capacity [11,12,13]. Against this backdrop, exploring potential allelopathic interactions within forest understory systems may inform the development of sustainable plant disease management strategies.

Pine species release various volatile organic compounds (VOCs) from both their rhizosphere and aerial tissues. These can influence the structures and functional potential of surrounding microbial communities via soil-mediated pathways, while producing direct inhibitory effects on specific phytopathogens [14]. Prior scientific evidence has documented that pine-derived VOCs may suppress pathogen growth directly and may also be associated with shifts in rhizosphere microbial communities, including the enrichment of certain beneficial microorganisms [15]. In traditional Chinese medicine, the concept of “using aromatic substances to purify” implicitly points to the effect of aromatic compounds on dispelling impurities or harmful influences. It also implies the potential role of volatile compounds in disease defense. Building on these insights, this study hypothesized that *P. yunnanensis* may be associated with reduced incidence of root rot in *P. tunicoides*, potentially through the release of VOCs that may directly inhibit pathogens or be involved in modulating the rhizosphere microbial community.

To test this hypothesis and explore the potential processes associated with reduced root rot, this study first extracted and characterized the principal chemical constituents of *P*. *yunnanensis*-extracted volatile oils from roots and needles. Subsequently, in vitro experiments were conducted to systematically evaluate their antifungal activities against four major root rot pathogens of *P. tunicoides*, namely *Fusarium solani* (*F. solani*), *Fusarium oxysporum* (*F. oxysporum*), *Fusarium redolens* (*F. redolens*), and *Rhizoctonia solani* (*R. solani*) [3,16]. Furthermore, a pot experiment using pine-derived volatile treatment was conducted to partially mimic pine-derived volatile exposure under controlled pot conditions. In addition, metagenomic analysis was employed to comprehensively assess the role of pine-derived volatiles in modulating the rhizosphere soil microbiome of *P. tunicoides*. The present study seeks to explore the ecological processes potentially responsible for disease suppression in forests of *P. yunnanensis*, aiming to provide a theoretical basis for environmentally sustainable management of root rot in *P. tunicoides* and offer scientific support for developing agroforestry-based cultivation systems for medicinal plants.

## 2. Results

### 2.1. Antifungal Activity of Volatile Oils

#### 2.1.1. Extraction and Chemical Composition of Volatile Oils

This study comprehensively characterized the chemical profiles of the extracted volatile oils, with the identification of the major constituents shown in Table 1. Turpentine (NEO) derived from the roots of *P. yunnanensis* was rich in α-pinene (45.50%) and longifolene (28.20%), whereas pine needle oil (OEO) was primarily composed of α-terpinyl acetate (75.13%). Additional chemical components are detailed in Supplementary Tables S3 and S4.

#### 2.1.2. Antifungal Activity Based on Oxford Cup Assay

**Figure 1:**
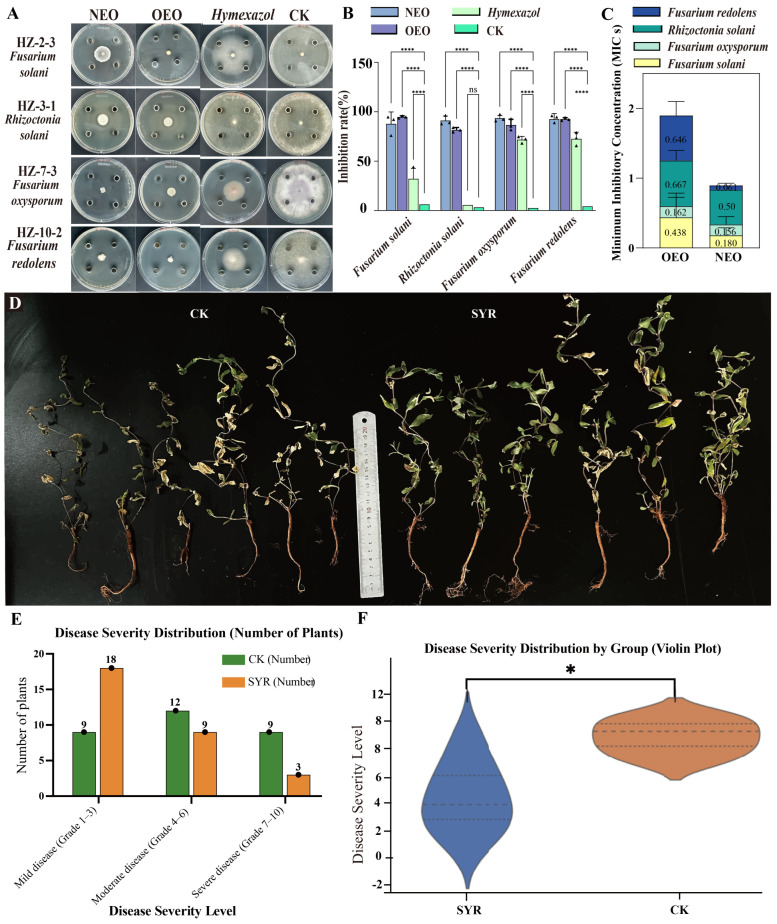
Effects of *P. yunnanensis*-derived volatile oils on root rot pathogens and disease severity in *P. tunicoides*. (**A**): Oxford cup assay for the antifungal activity of NEO and OEO against four root rot pathogens. Hymexazol (MZ) was used as the positive control, and 1-DMSO-T solution (CK) as the solvent negative control; (**B**): Quantitative analysis of inhibition rates obtained from the Oxford cup assay. Data represent three independent replicates (two-way ANOVA followed by Tukey’s multiple-comparison test. ns, not significant; **** *p* < 0.0001); (**C**): MICs of NEO and OEO against the four root rot pathogens. Values are presented as means ± SD from three independent replicates; (**D**): Phenotypic comparison of *P. tunicoides* plants following pathogen inoculation. CK: water-treated control; SYR: turpentine-treated group; (**E**): Distribution of disease severity grades in the CK and SYR groups. Disease severity was classified as mild (grades 1–3), moderate (grades 4–6), and severe (grades 7–10); (**F**): Violin plot illustrating the distribution of disease severity grades in the CK and SYR groups (Tukey’s test, * *p* < 0.05).

At a concentration of 50 mg·mL^−1^, both NEO and OEO exhibited varying degrees of inhibitory effects against all four tested pathogens in the Oxford cup assay (Figure 1A). Specifically, NEO showed preliminary growth-inhibitory effects against the tested fungal pathogens under the current in vitro conditions. with mean inhibition rates of 87.84%, 91.14%, 93.53%, and 92.57% against *F. solani*, *R. solani*, *F. oxysporum*, and *F. redolens*, respectively. OEO also demonstrated substantial inhibitory effects, with mean inhibition rates of 94.71%, 81.65%, 86.72%, and 92.51% against the same pathogens. In contrast, the chemical fungicide hymexazol showed markedly lower inhibitory efficacy, with mean inhibition rates of 32.82%, 5.70%, 71.79%, and 72.68%, respectively. The CK group showed only negligible inhibitory activity against the tested pathogens, with inhibition rates ranging from 2.61% to 6.25%. Both NEO and OEO significantly suppressed pathogen growth compared with those in the CK group (all *p* < 0.05), and their overall antifungal activity outperformed that of hymexazol (Table S7).

#### 2.1.3. MICs of Volatile Oils

The MIC values were calculated to evaluate the antifungal activity of two pine-extracted volatile oils against four fungal pathogens associated with root rot in *P. tunicoides*. Consequently, both oils exhibited inhibitory effects, revealing variations in MIC values among different fungal species (Figure 1C; Table S5). NEO exhibited MIC values of 0.06 ± 0.03, 0.16 ± 0.11, 0.18 ± 0.11, and 0.50 ± 0.00 mg·mL^−1^ against *F. redolens*, *F. oxysporum*, *F. solani*, and *R. solani*, respectively. On the contrary, OEO showed a MIC value of 0.16 ± 0.13 mg·mL^−1^ against *F. oxysporum*, and higher MIC values against *F. solani*, *R. solani*, and *F. redolens*. Detailed MIC results are provided in Table S5.

#### 2.1.4. Disease Severity Assessment

The effect of turpentine on disease severity was evaluated using a root rot model induced by *F. oxysporum* under the tested pot conditions. Therefore, the pot experiment results should be interpreted within the context of this representative pathogen system. Turpentine treatment was associated with reduced disease severity in *P. tunicoides* (Figure 1D). Disease severity in *P. tunicoides* was assessed using a modified 1–10 visual root rot rating scale adapted from the root rot severity classification proposed by Awodele et al. [17]. The detailed symptom descriptions and scoring criteria for each grade are shown in Supplementary Table S8. Among these, grades 1–3 are defined as mild infection (limited or localized symptoms), grades 4–6 as moderate (expanded lesions with partial functional impairment), and grades 7–10 as severe (extensive damage, often leading to severe growth inhibition or plant death).

As shown in Table 2, the estimated disease severity was lower in the SYR treatment than in CK. The disease index decreased from 51.5% in CK to 35.5% in SYR. In the SYR group, disease severity was predominantly distributed within the mild category (60%) (Figure 1E). In contrast, the CK group exhibited a more even distribution, with notably higher proportions of moderate (40%) and severe (30%) disease. Specifically, the proportion of severe cases in the SYR group was only 10%, substantially lower than that observed in the CK group (30%). Conversely, the proportion of mild cases was markedly higher in the SYR group (60% vs. 30%). In addition, the SYR group also showed a modest reduction in moderate disease incidence compared with the CK group (30% vs. 40%). These differences were further visualized using violin plots (Figure 1F). Statistical comparisons between treatments were performed using Tukey’s multiple comparison test, which indicated a statistically significant difference between the CK and SYR groups (*p* < 0.05). Overall, the SYR treatment reduced the occurrence of moderate and severe diseases. A shift in disease severity distribution was observed compared with the control.

### 2.2. Metagenomic Sequencing Results and Analysis

#### 2.2.1. Microbial Composition in the CK and SYR Groups

**Figure 2:**
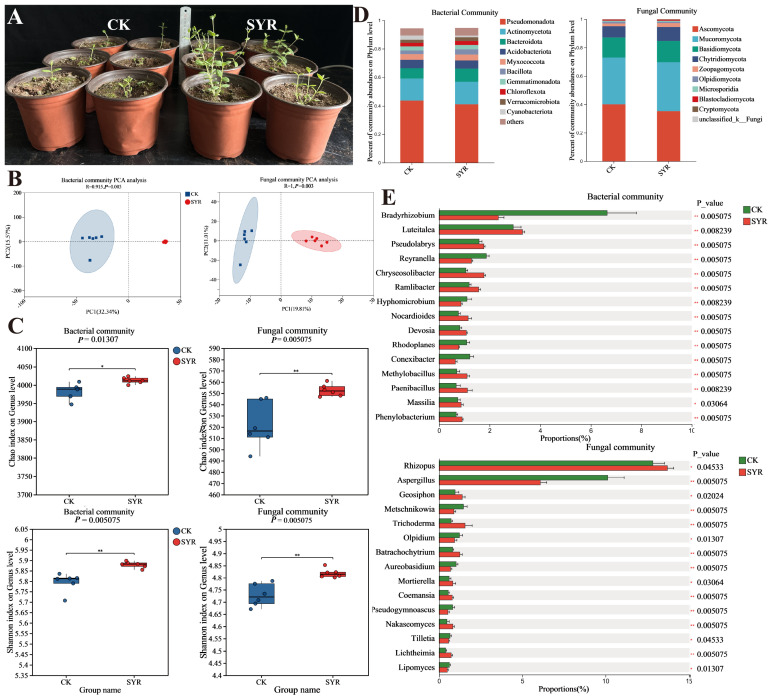
Alterations in plant growth and rhizosphere microbial communities following turpentine treatment. (**A**): Representative photographs of plants under water treatment (CK) and turpentine treatment (SYR); (**B**): Principal component analysis (PCA) of bacterial and fungal communities showing clear separation between the CK and SYR groups; (**C**): Alpha-diversity of bacterial and fungal communities based on Chao1 richness and Shannon diversity indices; (**D**): Relative abundance of dominant bacterial and fungal phyla in the CK and SYR groups; (**E**): Differentially abundant bacterial and fungal genera between the CK and SYR treatments. (* *p* < 0.05; ** *p* < 0.01).

In the pot experiment, turpentine application led to a clear shift in the phenotype of *P. tunicoides* (Figure 2A). Compared with the CK group, the SYR group showed a visually higher plant height, more branching, and greater biomass. Metagenomic sequencing identified a total of 6799 bacterial taxa and 256 fungal taxa across 12 libraries (six biological replicates per treatment), providing a robust dataset for assessing the effects of turpentine on the structural and functional properties of the rhizosphere microbial community. PCA revealed a clear separation between CK and SYR samples, indicating pronounced differences in microbial community structure (Figure 2B). For bacterial communities, the first and second principal components (PC1 and PC2) accounted for 32.34% and 15.57% of the total variance, respectively (47.91% combined). In fungal communities, PC1 and PC2 explained 19.81% and 11.01% of the variance, representing 30.82% of the total variation. This separation was further supported statistically by PERMANOVA (bacteria: *R* = 0.915, *p* = 0.003; fungi: *R* = 1.000, *p* = 0.003), indicating significant differences in microbial community structure between groups. However, due to the experimental design, we cannot fully exclude the possibility that residual volatiles directly inhibited pathogen growth in the pot experiment.

Comparative analysis of α-diversity metrics (Figure 2C) showed that the SYR group had higher Chao1 richness (*p* = 0.01307) and Shannon diversity index (*p* = 0.005075) compared with the CK group, suggesting significantly greater bacterial richness and diversity in the former group. A similar pattern was observed for fungal communities, where both the Chao1 and Shannon indices were significantly elevated in the SYR group. Altogether, turpentine treatment was associated with changes in both bacterial and fungal community composition in the rhizosphere of *P. tunicoides*, with increased taxonomic richness and diversity observed after treatment.

Figure 2D displays the relative abundance of bacterial and fungal communities at the phylum level in the CK and SYR groups. Within the bacterial community, *Pseudomonadota* and *Actinomycetota* dominated both treatments. The relative abundance of *Bacteroidota* was elevated in the SYR group compared with the CK group. For the fungal community, *Ascomycota* and *Mucoromycota* were the predominant phyla across both treatments. Notably, the SYR group exhibited higher relative abundances of *Basidiomycota* and *Chytridiomycota* than the CK group. One-way analysis of variance with Tukey–Kramer post hoc testing (Figure 2E) revealed significant differences at the genus level. In the bacterial community, the SYR treatment significantly enriched several taxa commonly associated with beneficial ecological functions, including *Pseudolabrys*, *Chryseosolibacter*, *Massilia*, *Paenibacillus*, *Methylobacillus*, and *Phenylobacterium*. Similarly, in the fungal community, the SYR group exhibited higher relative abundances of several genera, such as *Rhizopus*, *Geosiphon*, *Trichoderma*, *Batrachochytrium*, *Mortierella*, *Coemansia*, *Nakaseomyces*, and *Lichtheimia*.

#### 2.2.2. Rhizosphere Microbial Co-Occurrence Networks and Culturable Antagonistic Bacteria Under SYR Treatment in *P. tunicoides*

**Figure 3:**
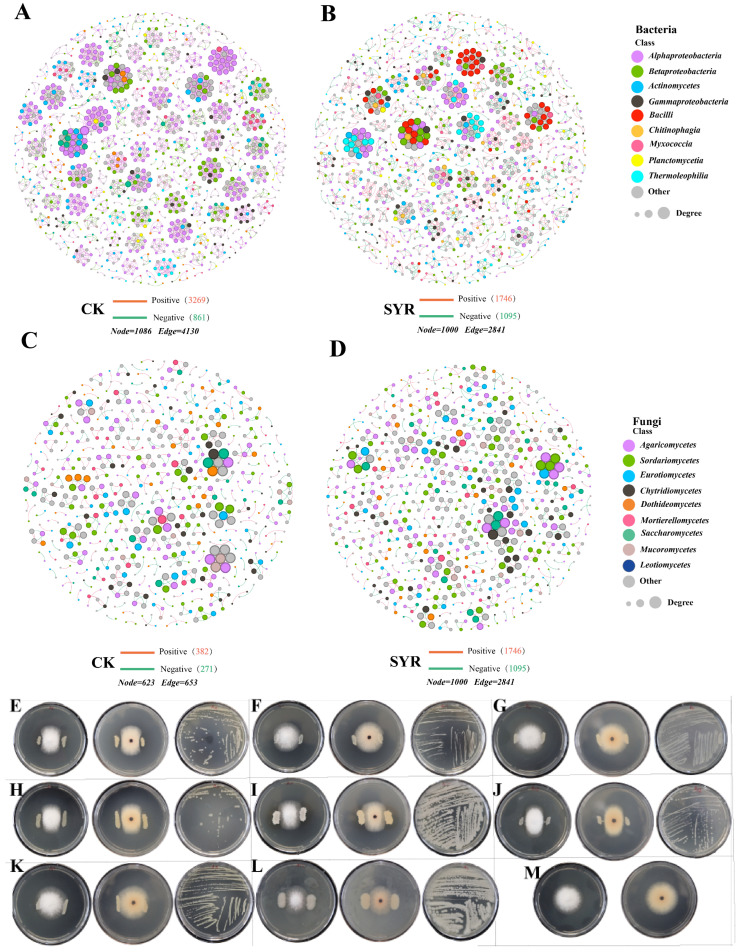
Rhizosphere microbial co-occurrence networks and antagonistic activity of culturable bacterial isolates under CK and SYR treatments. (**A**,**B**): Co-occurrence networks of bacterial communities in the CK (**A**) and SYR (**B**) groups; (**C**,**D**): Co-occurrence networks of fungal communities in the CK (**C**) and SYR (**D**) groups. (**E**–**L**) Antagonistic activity of bacterial isolates against *F. oxysporum* ((**E**): *Streptomyces nozoeensis*; (**F**): *Streptomyces cinereus*; (**G**): *Cupriavidus campinensis*; (**H**): *Streptomyces spororaveus*; (**I**): *Bacillus pallens*; (**J**): *Paenibacillus polymyxa*; (**K**): *Paenarthrobacter nicotinovorans*; (**L**): *Bacillus weihenstephanensis*). (**M**) Control plate inoculated only with *F. oxysporum*. NOTE: Nodes represent microbial taxa, and they are colored according to class-level taxonomy. Node size indicates the degree of connectivity. Edge colors denote positive (orange) or negative (green) correlations. Network statistics (node and edge numbers) are indicated below each panel. The networks were constructed based on statistically significant Spearman correlations among core OTUs and should be interpreted only as exploratory visualizations of potential co-occurrence patterns.

This study further constructed the co-occurrence networks of bacterial and fungal communities, as depicted in Figure 3, to investigate the possible impact of turpentine application on microbial correlations in the rhizosphere. Pronounced inter-group differences were noticed in both network topology and interaction patterns. In the bacterial co-occurrence networks (Figure 3A,B), each network consists of nodes (i.e., OTUs) and edges (i.e., correlations between OTUs). Nodes are color-coded by taxonomic affiliation, including *Alphaproteobacteria*, *Betaproteobacteria*, *Actinomycetes*, *Gammaproteobacteria*, *Bacilli*, *Chitinophagia*, *Myxococcia*, *Planctomycetia*, *Thermoleophilia*, and other groups. Node size reflects degree centrality, while edge colors denote positive (orange) or negative (green) correlations. In the network of the CK group (Figure 3A), a total of 1086 nodes and 4130 edges were identified, with an average degree of 7.61. Among these correlations, 3269 were positive and 861 were negative, with a modularity index of 0.976 (Table S6). In contrast, the network of the SYR group (Figure 3B) contained 1000 nodes and 2841 edges, with a reduced average degree of 5.68. Positive correlations decreased to 1746, whereas negative correlations increased to 1095, accompanied by a slightly higher modularity index of 0.979 (Table S6). From a compositional perspective, taxa such as *Alphaproteobacteria*, *Actinomycetes*, *Gammaproteobacteria*, and *Bacilli* dominated the bacterial networks in both groups. In particular, many taxa affiliated with *Bacilli*, *Betaproteobacteria*, and *Actinomycetes* showed relatively high connectivity in the SYR group. This pattern suggests that volatile oil treatment may be associated with changes in the network positions of these taxa. Notably, Bacilli were especially prominent in the SYR group, while other communities, such as *Chitinophagia* and *Myxococcia,* also showed increased representation.

Fungal network analysis further indicated that the network of the CK group was comparatively smaller and less complex (Figure 3C), comprising 623 nodes and 653 edges, including 382 positive and 271 negative correlations. Negative edges accounted for 41.5% of the total, with an average degree of 2.10 and a modularity index of 0.991 (Table S6). In contrast, the network of the SYR group exhibited a clear expansion in scale and connectivity (Figure 3D), consisting of 697 nodes and 787 edges, with an increased average degree of 2.26. Among these, 426 edges were positive and 361 were negative, and the modularity index slightly increased to 0.992 (Table S6). Both networks shared identical topological features in terms of average path length (1), network diameter (1), and clustering coefficient (1). Overall, turpentine treatment increased the number of nodes, edges, and average degree in the fungal network, while slightly decreasing network density and elevating the proportion of negative correlations. These findings implied inter-group differences in the fungal and bacterial co-occurrence patterns, suggesting that turpentine treatment may be associated with changes in rhizosphere microbial interaction networks. In addition, in terms of taxonomic composition, *Agaricomycetes*, *Sordariomycetes*, and *Dothideomycetes* were core fungal groups in both groups. However, lower-abundance taxa such as *Eurotiomycetes* and *Chytridiomycetes* showed notable expansion in the SYR group.

To further support the presence of potentially beneficial microorganisms in the SYR-treated rhizosphere, culturable bacterial isolates were obtained and evaluated for antagonistic activity against *F. oxysporum*. Compared with the control group (Figure 3M), Eight isolates belonging to *Bacillus*, *Streptomyces*, *Cupriavidus*, *Paenibacillus*, and *Paenarthrobacter* showed inhibitory effects on *F. oxysporum* growth in plate confrontation assays, with inhibition zones of different sizes (Figure 3E–L). The above results suggest that SYR treatment may promote the enrichment of culturable antagonistic bacteria with potential biocontrol functions in the rhizosphere environment.

#### 2.2.3. Effects of Turpentine on the Potential Functional Profiles of the Rhizosphere Microbiome

**Figure 4:**
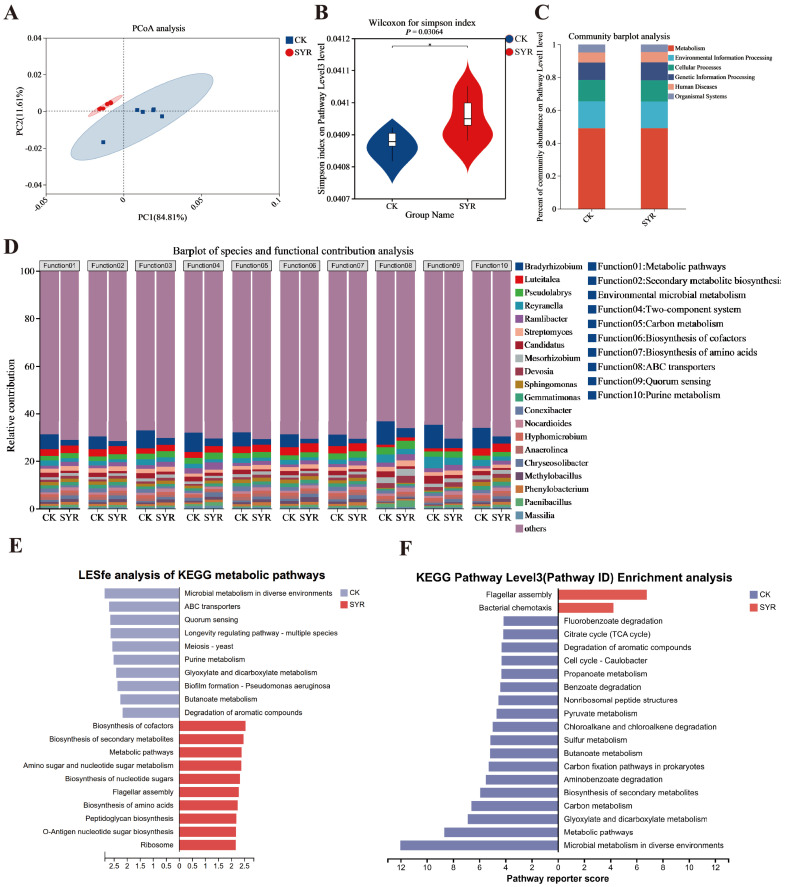
Functional profiling of rhizosphere microbial communities under CK and SYR treatments based on KEGG annotations. (**A**): Principal coordinates analysis (PCoA) of microbial functional profiles based on KEGG level 3 pathways; (**B**): comparison of functional diversity between groups using the Simpson index (Wilcoxon test); (**C**): relative abundance of KEGG functional categories at the class level in the two groups; (**D**): contribution of dominant genera to major functional pathways; (**E**): differential KEGG metabolic pathways identified by LEfSe analysis between groups; (**F**): Enrichment analysis of KEGG level 3 pathways showing differentially enriched predicted functional pathways.

To further explore potential differences in the predicted functional profiles of the rhizosphere microbiome of *P. tunicoides* under turpentine treatment, functional diversity assessment and KEGG pathway enrichment analyses were conducted based on the data of metagenomic sequencing. Principal coordinates analysis (PCoA) revealed a clear separation between CK and SYR samples along PC1, which explained 84.81% of the total variation (Figure 4A). At the KEGG pathway level 3, analysis of functional diversity within microbial communities showed a significantly higher Simpson diversity in the SYR group compared with the CK group (Figure 4B; *p* = 0.03064). This result suggests that turpentine treatment was associated with differences in the predicted functional profiles of the rhizosphere microbiome. Additionally, functional annotation results (Figure 4C) indicated that major pathways were consistently enriched in both the SYR and CK groups, including metabolic pathways, biosynthesis of secondary metabolites, environmental microbial metabolism, and two-component systems.

Based on the functional contribution analysis (Figure 4D), *Bradyrhizobium*, *Luteitalea*, *Pseudolabrys*, *Reyranella*, *Ramlibacter*, and *Streptomyces* were the contributors to functional potential in both the SYR and CK groups. Compared with the CK group, the SYR group showed general reduction in the relative contribution of *Bradyrhizobium* to key functional pathways, but marked enrichment in taxa such as *Streptomyces*, *Methylobacillus*, and *Paenibacillus*. To improve data clarity and visualization, the corresponding quantitative results are presented in the form of bar plots with statistical analysis, as illustrated in Supplementary Figure S1.

KEGG-based functional prediction further revealed substantial differences in metabolic potential between the two treatments (Figure 4E,F). As evidenced by the LEfSe results, the CK group was primarily enriched in pathways associated with basal metabolism and organic matter turnover, including microbial metabolism in diverse environments, ATP-binding cassette transporters, quorum sensing, glyoxylate and dicarboxylate metabolism, butanoate metabolism, and degradation of aromatic compounds. In contrast, the SYR treatment was significantly enriched in pathways related to biosynthetic and cellular processes, such as biosynthesis of cofactors, biosynthesis of secondary metabolites, amino sugar and nucleotide sugar metabolism, biosynthesis of amino acids, peptidoglycan biosynthesis, ribosome, and flagellar assembly (Figure 4E).

The pathway enrichment at KEGG level 3 further substantiated these functional distinctions (Figure 4F). The CK group exhibited higher reporter scores in pathways such as microbial metabolism in diverse environments, metabolic pathways, carbon metabolism, and glyoxylate and dicarboxylate metabolism. By contrast, the SYR group was significantly enriched in pathways associated with microbial motility and environmental sensing, including flagellar assembly and bacterial chemotaxis. Overall, SYR treatment may enhance the motility of rhizosphere microorganisms and improve their capacity to perceive and respond to environmental changes.

## 3. Discussion

### 3.1. Direct Inhibitory Effect of Pine-Extracted Volatile Oils on Major Pathogens of P. tunicoides

*P. tunicoides*, an endangered, regionally endemic medicinal plant in southwestern China, has long been valued in traditional Chinese medicine for its notable pharmacological properties. However, frequent outbreaks of root rot severely constrain the large-scale production of this medicinal plant under cultivation conditions. Beyond ecological and human health risks, the prolonged use of chemical fungicides may also promote the development of resistance in pathogenic fungi, resulting in diminished control efficacy [18]. In this context, pine-extracted volatile oils, given their strong antifungal activity, rapid degradability, and low ecological persistence, have been accepted as environmentally compatible antimicrobial agents [19,20]. It has been demonstrated that volatile oils possess a multi-target antifungal mechanism of action [21]. Initially, they can interact with fungal cell membrane proteins, disrupting the phospholipid bilayer and increasing membrane permeability. It may facilitate further penetration of active constituents into cells to induce structural damage and cell death. Furthermore, upon entering the cells, these active components can interfere with several critical enzyme systems of the pathogens to impede their normal physiological and metabolic processes [22]. Moreover, volatile oils can inhibit the mycelial growth of pathogenic fungi, consequently preventing their colonization and proliferation on plant surfaces [23,24,25]. In this study, in planta pot experiments (greenhouse conditions) provided preliminary evidence that treatment with pine-extracted volatile oils reduced disease symptoms in *P. tunicoides* infected with *F. oxysporum* (Figure 1D), highlighting their potential for managing root rot in this species.

To elucidate the chemical basis underlying the antifungal activity of pine-extracted volatile oils, the roots and needles of *P. yunnanensis* were subjected to steam distillation, yielding turpentine (NEO) and pine needle oil (OEO), respectively. Subsequent characterization of their chemical constituents using GC-MS unveiled that NEO was primarily composed of α-pinene (45.50%) and longifolene (28.20%), whereas OEO was dominated by α-terpinyl acetate (75.13%) and β-terpinyl acetate (7.24%). These monoterpenes and sesquiterpenes are widely recognized for their broad-spectrum antimicrobial properties [26,27]. Semerdjieva et al. demonstrated that α-pinene was the predominant constituent in the volatile oils derived from four *Pinus* species (*P. heldreichii*, *P. peuce*, *P. sylvestris*, and *P. nigra*). Furthermore, this compound exhibited significant inhibitory activities against various pathogens, including the fungi *Alternaria alternata*, *Botrytis cinerea*, *Diaporthe nobili*, *F. oxysporum*, *R. solani*, as well as the oomycete “*Phytophthora cryptogea*” [28]. Islamiati et al. found that *Streptomyces* sp. GMR22 was capable of producing several secondary metabolites, such as VOCs. This study indicated that turpentine oil also contained longifolene, which has been previously reported to exhibit inhibitory effects against plant pathogenic fungi [29]. Notably, longifolene emerged as the most prominent antifungal VOC produced by *Streptomyces* sp. GMR22, demonstrating the ability to effectively suppress the growth of *F. oxysporum* and *G. boninense* [30]. Taken together, the enrichment of such terpenoids may serve as a key factor underlying the antifungal efficacy of pine-extracted volatile oils.

To further verify the above inference, Oxford Cup assays were conducted to evaluate the direct antifungal activity of pine-extracted volatile oils against the four pathogens of *P. tunicoides*. Consequently, NEO achieved mean inhibition rates of 87.84%, 91.14%, 93.53%, and 92.57% against *F. solani*, *R. solani*, *F. oxysporum*, and *F. redolens*, respectively. Notably, the inhibition rates against *R. solani*, *F. oxysporum*, and *F. redolens* exceeded 90%, highlighting strong antifungal activities against the tested root rot-associated pathogens. OEO also exhibited significant inhibitory effects, with mean inhibition rates of 94.71%, 81.65%, 86.72%, and 92.51% against the same panel of pathogens. Accordingly, the MICs of the volatile oils against these pathogens were determined to provide a quantitative basis for subsequent pot experiments. Based on the criteria proposed by Madbouly et al. [31], the antifungal activity can be classified as strong (MIC < 100 μg·mL^−1^), moderate (100–625 μg·mL^−1^), and weak (>625 μg·mL^−1^). Within this framework, both NEO and OEO exhibited varying degrees of antifungal activity against the tested pathogens (Table S5). Notably, NEO showed strong inhibition against *F. redolens*, with an MIC of 0.06 ± 0.03 mg·mL^−1^ (approximately 61 μg·mL^−1^). In short, consistent with previous studies reporting the antimicrobial potential of pine-derived products [32], both pine-extracted volatile oils exhibited antifungal activities against all tested pathogens, despite variations in inter-species inhibitory efficacy. Overall, NEO and OEO showed inhibitory effects on fungal growth in both the Oxford cup assay and MIC determination, suggesting their antifungal potential. Nevertheless, inhibition zones in the Oxford cup assay may be affected by the volatility, hydrophobicity, and diffusion properties of essential oil components, while OD595-based MIC determination for filamentous fungi may be influenced by hyphal aggregation and growth morphology. Thus, these results should be interpreted as relative indicators of antifungal activity under the present conditions. Further assays, including spore germination, mycelial biomass, MFC, and time-kill analyses, are needed to more precisely define the antifungal efficacy and action properties of these volatile oils.

Collectively, the two pine-extracted volatile oils exhibited pronounced direct inhibitory effects against the root rot-associated pathogens of *P. tunicoides* identified in a previous study [3]. These findings provide experimental evidence that pine-derived volatiles can suppress major root rot pathogens under controlled conditions, thereby offering a plausible partial explanation for the lower incidence of root rot observed in *P. tunicoides* populations growing beneath pine canopies.

This study represents a preliminary in vivo validation using a single pathogen (*F. oxysporum*) and one biologically effective concentration. In the pot experiment, SYR was applied as a DMSO/Tween 80 formulation through a slow-release setup, without a matched vehicle control or quantification of volatile constituents in the soil, rhizosphere, and headspace. Moreover, the GC-MS results represent putative chemical profiles based on NIST library matching and relative peak area normalization, rather than confirmed quantitative characterization. Disease severity was also assessed mainly by visual symptom scoring and disease grade distribution. Therefore, the observed effects on plant growth, disease severity, and rhizosphere communities should be interpreted cautiously as responses to the SYR formulation under the tested conditions. Future studies incorporating multi-pathogen and dose–response designs, vehicle controls, volatile monitoring, validated chemical characterization, growth measurements, mortality records, and blinded scoring would improve the robustness of these findings.

### 3.2. Effects of Pine-Extracted Volatile Oils on Root Rot Resistance and Rhizosphere Microbial Community Structure of P. tunicoides

The preceding results demonstrated that pine-derived volatile oils exhibit potent direct inhibitory effects against multiple root rot pathogens of *P. tunicoides*. Beyond this direct antifungal activity, these plant-derived volatiles may be associated with ecological changes in the rhizosphere microbial community composition. To examine this ecological response, a comparative metagenomic analysis was conducted using rhizosphere soils from the SYR and CK groups. PCoA revealed a clear separation between CK and SYR samples, indicating that volatile oil treatment was associated with shifts in rhizosphere microbial community structure. However, these structural differences reflect community-level sensitivity to environmental perturbation and do not provide evidence that such shifts are required for disease suppression. Consistently, α-diversity analysis showed that richness and diversity indices, including Chao1 and Shannon, were significantly higher in the SYR group than in the CK group (all *p* < 0.05). Therefore, pine-derived volatile compounds may be associated with changes in the rhizosphere microbiome. As illustrated in Figure 2E, differences in microbial composition between SYR and CK samples were examined to further characterize these shifts. In the bacterial community, the SYR treatment significantly enriched several taxa potentially associated with plant growth promotion and antagonistic activity, including *Paenibacillus* and *Methylobacillus*. Notably, members of the genus *Paenibacillus* are widely recognized for their biocontrol potential. Members of *Paenibacillus* have been reported to produce a range of antimicrobial compounds that suppress pathogenic microorganisms and secrete cell wall-degrading enzymes such as lysozymes and chitinases, which can disrupt the structural integrity of fungal cell walls [33,34,35,36] and produce antibacterial compounds such as lipopeptides, including surfactin, polymyxin, and pelgipeptin [37]. Furthermore, these bacteria are capable of eliciting plant defense responses and have been reported to be associated with induced systemic resistance (ISR) in host plants against pathogen invasion [38,39]. Within the fungal community, the SYR treatment likewise resulted in a significant enrichment of beneficial taxa, particularly those belonging to the genus *Trichoderma*. Species of *Trichoderma* are well-recognized soil-borne antagonistic fungi that have been reported to suppress plant pathogens through multiple mechanisms [40]. These include the production of diverse volatile and non-volatile secondary metabolites with antifungal activities [41], as well as the secretion of cell wall-degrading enzymes (e.g., chitinases, glucanases, and proteases) that disrupt the structural integrity of pathogenic fungi [42]. Moreover, *Trichoderma* can rapidly colonize the rhizosphere and may colonize the rhizosphere and compete for ecological niches through competition for nutrients and ecological niches. They are also known to activate systemic resistance, further strengthening host defense capacity [43]. Additionally, the SYR group also showed enrichment of other potentially beneficial microorganisms, such as *Geosiphon*, in addition to *Trichoderma*. *Geosiphon* is strongly associated with soil nutrient cycling, nitrogen fixation and ecosystem stability [44]. Studies have shown that *Geosiphon*, through forming endosymbiotic associations with nitrogen-fixing cyanobacteria, provides host plants with bioavailable nitrogen and water, potentially enhancing their innate defense capacity [45].

Previous studies have demonstrated that some plants (such as *Pinus* species) synthesize and release a diverse array of volatile organic compounds (VOCs) both constitutively and in response to biotic and abiotic stresses. These volatiles can function as chemical cues or signaling molecules mediating intra- and interspecific plant interactions [46]. Zhou [47] et al. demonstrated that potato-onion volatile organic compounds (VOCs), specifically the active molecule dipropyl disulfide, interactively stimulated the growth of adjacent tomato plants. This interspecific communication was mediated by induced alterations in tomato root exudates, which indirectly recruited plant growth-promoting bacteria, such as *Pseudomonas* and *Bacillus* spp., to restructure the rhizosphere microbiota. Ji [12] et al. investigated the interactions driving disease suppression in a *Pinus koraiensis*–*Panax ginseng* system. The study found that the incidence of *Alternaria* leaf spot on *P. ginseng* was significantly reduced under *P. koraiensis* forests. Mechanistically, endo-borneol from *P. koraiensis* needle leachates directly induce resistance in neighboring *P. ginseng* plants. Concurrently, it indirectly recruited beneficial rhizospheric microbes by promoting the secretion of ginsenosides of *P. ginseng*, thus triggering induced systemic resistance (ISR).

Overall, the volatile oil treatment was associated with marked shifts in the rhizosphere microbial community of *P. tunicoides*, accompanied by increased relative abundances of several beneficial microbes, such as *Paenibacillus* and *Trichoderma*. Consequently, we hypothesize that pine volatile oil may stimulate the release of specific root-derived chemoattractants, thereby potentially facilitating the recruitment or enrichment of beneficial microbes, which may be linked to reduced disease severity. This microbiome shifts in the rhizosphere microbiome may act in concert with the direct antifungal activity described above and may be associated with the observed reduction in disease severity in *P. tunicoides* after root rot pathogen inoculation. However, this study has several limitations. Specifically, the current study did not determine whether turpentine treatment alters the production of specific signaling compounds by *P. tunicoides*, or whether such changes are associated with the recruitment or enrichment of bacterial taxa previously linked to plant-beneficial traits. We will validate this biological process through dual-culture antagonism, biofilm formation, and chemotaxis assays. With these attempts, we may systematically interpret the relative contributions of direct chemical inhibition versus plant-mediated microbial regulation to disease suppression.

### 3.3. The Effect of Pine-Extracted Volatile Oils on Reshaping the Co-Occurrence Network Structure of the Rhizosphere Microbiome in P. tunicoides

This study further constructed co-occurrence networks for each treatment group, aiming to explore the potential influence of pine-derived volatile treatment on microbial co-occurrence patterns in the rhizosphere microbiome of *P. tunicoides* and to identify key taxa under different conditions. In our study, the post-treatment proportion of negative correlations increased markedly in the SYR group (from 21% to 39%) in the bacterial network whereas the total number of edges (4130 → 2841) and the average degree (7.61 → 5.68) both declined (Table S6). Despite this reduction in overall connectivity, the modularity index of the SYR network remained high (0.979 > 0.4). It may imply that, under volatile oil treatment, the microbial community tends to organize into multiple relatively independent functional modules. Within each module, taxa showed relatively dense correlation-based associations, yet with potentially reduced connectivity between modules [48]. Meanwhile, negative correlations were increased in the SYR experimental group. Studies have shown that such highly modular organization, together with increased negative correlations, can confine the effects of environmental perturbations within specific modules, thereby preventing disturbances from propagating across the entire network [49,50]. Conversely, the fungal network exhibited an increase in both node number and edge number in the SYR group (697 nodes and 787 edges), accompanied by an elevated average degree, indicating greater network complexity and connectivity. Prior studies have reported that microbial networks with higher complexity and connectivity are generally more stable, highly resilient to environmental disturbances, and more effective in defending against pathogen invasion [51,52]. This may be explained by the typically more frequent and intricate interspecific interactions in highly connected microbial communities, indicating intensified competition for ecological niches and resources. Such dynamics can restrict pathogen establishment, while simultaneously promoting the activation of plant immune responses [53]. Regarding key microbial taxa, notable shifts were observed in the bacterial community. In the CK group, *Alphaproteobacteria* functioned as the principal network hubs, whereas the network from SYR samples indicated *Bacilli* and *Actinobacteria* as the major putative keystone hub taxa, both with relatively high centrality within the network. These patterns are largely consistent with the taxonomic shifts identified in the metagenomic analysis of community composition. Reportedly, members of *Bacilli* and *Actinomycetes* can promote plant growth and enhance disease resistance through multiple mechanisms, including phytohormone production, mineral solubilization, organic matter decomposition, and the synthesis of antimicrobial compounds that suppress pathogenic microorganisms. In disease management, they can not only secrete various antibacterial compounds, such as lipopeptide antibiotics, effectively inhibiting the growth of phytopathogenic fungi and bacteria, but also produce chitinases that directly degrade fungal cell walls to kill fungal pathogens [54]. On the other hand, actinomycetes, as a group of Gram-positive bacteria, also exhibit substantial biocontrol potential. They are capable of secreting various antibiotics and hydrolytic enzymes, such as chitinases, to effectively suppress plant diseases. Given these, they may be promising biological control agents, contributing to a significant reduction in the use of chemical pesticides in agricultural production [55]. In the fungal community, *Agaricomycetes*, *Sordariomycetes*, and *Dothideomycetes* remained core taxa in both groups. However, SYR treatment was also associated with the expansion of lower-abundance classes such as *Eurotiomycetes* and *Chytridiomycetes*. This shift suggests that volatile oil treatment may be associated with changes in the preexisting rhizosphere microbial correlation-based co-occurrence patterns, accompanied by altered relative contributions or network positions of potentially functional or antagonistic taxa, thereby indicating a possible reorganization of the functional potential of the rhizosphere microbiome.

In this study, co-occurrence network analysis was mainly used to explore changes in potential association patterns within the rhizosphere microbial community under different treatment conditions. Because the analysis was based on Spearman correlations, involved a limited number of biological replicates per group (*n* = 6), and microbiome data are inherently compositional, the observed network links should be interpreted as correlation-level co-occurrence signals. In future work, we will increase the sample size, incorporate absolute abundance and time-series data, and apply network inference methods that are more suitable for compositional data, such as SparCC and SPIEC-EASI, to further evaluate the robustness and biological significance of these co-occurrence associations.

### 3.4. Effects of Volatile Oil Treatment on the Potential Functional Profiles of the Rhizosphere Microbiome in P. tunicoides

Building upon the functional contribution analysis (Figure 4D), the potential functions of the rhizosphere microbiome in *P. tunicoides* were primarily associated with metabolic pathways, biosynthesis of secondary metabolites, and environmental microbial metabolism. In both the SYR and CK groups, *Bradyrhizobium*, *Luteitalea*, *Pseudolabrys*, *Reyranella*, *Ramlibacter*, and *Streptomyces* were identified as the primary contributors to these functional processes. However, compared with the CK group, the relative abundance of *Bradyrhizobium* declined in the SYR group, whereas taxa such as *Streptomyces*, *Methylobacillus*, and *Paenibacillus* were significantly enriched. This shift suggests that volatile oil treatment was associated with changes in microbial taxa related to predicted functional pathways, potentially reflecting differences in rhizosphere microbial functional potential and ecological adaptation. Furthermore, differential enrichment analyses on functional genes between treatments were performed to provide insights into the potential functional basis underlying this adaptive enrichment. Both KEGG and LEfSe analyses consistently showed that, in addition to metabolic pathways and secondary metabolite biosynthesis pathways, flagellar assembly and chemotaxis were significantly enriched in the SYR group.

Flagellar assembly refers to the intracellular process by which bacterial flagella are constructed and assembled. It forms the structural basis for bacterial chemotaxis, as the rotation of the flagellar motor determines movement patterns (e.g., swimming and tumbling), thereby enabling directional migration [56]. For plant growth-promoting rhizobacteria, successful colonization of the rhizosphere is a prerequisite for functional activity. Chemotaxis and flagellar motility have been proven to be key drivers of this colonization process [57]. Within the rhizosphere, gradients of nutrients generated by root exudates serve as chemical cues that are detected by bacterial chemoreceptors [58,59]. In response, bacteria achieve directed movement through flagellar rotation, allowing them to migrate toward and effectively colonize the root surface [60].

*Bacillus velezensis* has been demonstrated to recognize plant root exudates through multiple chemoreceptors and subsequently establish stable colonization on root surfaces [61]. Similarly, the chemotaxis systems of *Pseudomonas putida* KT2440 and *Bacillus amyloliquefaciens* SQR9 are capable of sensing diverse chemoattractants present in rhizosphere exudates, which may be associated with rhizosphere adaptation [62,63]. From a taxonomic perspective, *B. velezensis* and *B. amyloliquefaciens* are phylogenetically closely related members of the *B. subtilis* species complex and belong to the “operational group *B. amyloliquefaciens*”, their taxonomy and genomic features have been further clarified by phylogenomic and comparative genomic analyses [64,65,66]. Additionally, Almirón et al. reported that a plant growth-promoting *Bacillus* strain (VMY10), isolated from the tomato rhizosphere, harbors multiple flagellar assembly associated genes (e.g., *flh*, *fli*, *flg*, and *motAB*) and chemotaxis-related genes (e.g., *cheA*, *cheB*, *cheR*, *cheV*, *cheW*, and *cheY*). All these play critical roles in successful rhizosphere colonization [67]. Given the increased relative abundance of beneficial microbial taxa, such as *Paenibacillus* and *Streptomyces*, in the SYR group, we speculate that these bacterial taxa may be linked to enhanced functional potential related to flagellar assembly and chemotaxis, which may facilitate rhizosphere adaptation. However, although KEGG annotation and LEfSe analysis provide valuable insights into treatment-related differences in the functional potential of the rhizosphere microbiome, the biological activity of these predicted pathways remains to be verified. Further transcriptomic, biochemical, and phenotypic validation would be needed to evaluate the biological relevance of these metagenome-derived predictions.

## 4. Materials and Methods

### 4.1. Antifungal Assay of Volatile Oils

#### 4.1.1. Pathogen Sources

The four pathogenic strains, *F. solani*, *F. oxysporum*, *F. redolens*, and *R. solani*, were isolated from root rot-infected *P. tunicoides* by our Research Group [3,16], which were identified and preserved under laboratory conditions. Species identification was performed based on morphological characteristics combined with fungal rDNA-ITS and TEF-1α sequence analyses, as well as phylogenetic tree construction for species confirmation. Materials for volatile oil extraction: Pine materials used for volatile oil extraction were collected from *P. yunnanensis*. growing in the natural habitat of *P. tunicoides* in Jianchuan County, Dali Bai Autonomous Prefecture, Yunnan Province (a recognized geo-authentic production area for *P. tunicoides*). The botanical identity of the pine material was confirmed as *P. yunnanensis*. by Professor Chunxia Pu from Yunnan University of Chinese Traditional Medicine, who specializes in plant taxonomy and medicinal plant research. *P. yunnanensis* is the dominant pine species associated with the habitat of *P. tunicoides* in the study area. This species can be identified by its reddish-brown bark, needles usually arranged in fascicles of two, and ovoid to conical-ovoid cones that turn chestnut-brown at maturity. Turpentine (NEO) and pine needle oil (OEO) were derived from roots and needles of *P. yunnanensis*, respectively. The ecological association between *P. yunnanensis* forests and the reduced incidence of root rot in *P. tunicoides* provided the basis for our research hypothesis. Additionally, this section also provides source support for subsequent extraction of turpentine via distillation. All reagents and instruments used in this study are detailed in Supplementary Tables S1 and S2, respectively.

#### 4.1.2. Preparation of Pathogen Cultures and Spore Suspensions

Fungal strains were incubated on potato dextrose agar (PDA) plates at 25 °C in the dark for 4–7 days until abundant sporulation was observed. The spore suspension was collected after the culture surface was gently flooded with sterile distilled water. Following 7 days of incubation at 28 °C on a rotary shaker (DG-800, Beijing Dingguo Guosheng Instrument Co., Ltd., Beijing, China) (150 rpm) in potato dextrose broth (PDB), the cultures were filtered through four layers of sterile gauze to remove mycelial debris. The filtrate was then centrifuged at 4000 rpm for 10 min at 4 °C to collect the spores. After the resuspension of the resulting spore pellet in sterile distilled water, the spore concentration was adjusted to 1 × 10^6^ spores/mL using a hemocytometer (Shanghai Qiujing Medical Instrument Co., Ltd., Shanghai, China) for subsequent inoculation experiments.

#### 4.1.3. Extraction and Chemical Characterization of Volatile Oils

Volatile oils were extracted from pine roots and needles. For each sample type, 500 g of fresh materials were subjected to hydrodistillation using previously described methods [68]. The extraction was conducted for 6 h to extract three independent batches of *P. yunnanensis* root materials separately. The collected volatile oils were dried over anhydrous sodium sulfate (analytical grade, 99.0%; Tianjin Zhiyuan Chemical Reagent Co., Ltd., Tianjin, China) and then extracted with cyclohexane (analytical grade, 99.5%; Tianjin Zhiyuan Chemical Reagent Co., Ltd., Tianjin, China). The resulting extracts were transferred into amber glass vials and stored at 4 °C for subsequent analysis. Furthermore, gas chromatography-mass spectrometry (GC-MS) was performed to determine the chemical composition and relative abundance of the volatile oils. Gas chromatographic separation was performed on an Agilent 19091J-115 HP-5 column (Agilent Technologies, Santa Clara, CA, USA) (5% phenyl methyl siloxane; 50 m × 0.32 mm × 0.52 μm). The injector and flame ionization detector were both set at 250 °C. The oven temperature was programmed from 50 °C to 250 °C at 5 °C·min^−1^. The injection volume was 1 μL, with a split ratio of 20:1. The inlet pressure was maintained at 102.28 kPa, and the volumetric flow rate was 2.1 mL·min^−1^, using high-purity helium as the carrier gas. Mass spectrometric conditions were set as: electron impact ionization at 70 eV, scan range of 50–550 m/z, ion source temperature of 230 °C, and quadrupole temperature of 150 °C. The solvent delay time was set to 6 min. Quantification was performed using manual peak integration combined with relative peak area normalization, and compound identification was based on comparison with the NIST 20 mass spectral library [69].

#### 4.1.4. Oxford Cup Assay for Antifungal Activity of Volatile Oils [70]

The antifungal activity of volatile oils was evaluated using the modified Oxford cup method. In brief, volatile oils and hymexazol (Beijing Zhongke Biochemical Reagent Co., Ltd., Beijing, China) were separately dissolved in a mixed solvent system consisting of 1% (*v*/*v*) dimethyl sulfoxide (DMSO) (Shanghai Hengyuan Chemical Reagent Co., Ltd., Shanghai, China) and 0.1% (*v*/*v*) Tween 80 (1-DMSO-T) to obtain homogeneous suspensions. Before use, the suspensions were sterilized by filtration through 0.22 μm. Under aseptic conditions, PDA (20 mL per plate) was poured into Petri dishes and allowed to solidify. Mycelial plugs (5 mm) from actively growing colonies were centrally inoculated onto the plates. Four Oxford cups were positioned equidistantly (25 mm) around the inoculum. After that, 200 μL of the filtered volatile oil or hymexazol suspension (both at 50 mg·mL^−1^) was added into each cup. In this assay, the 1-DMSO-T solution and hymexazol served as the negative control and the positive control, respectively. Each treatment was carried out with three replicates and cultured in a microbiological incubator at a constant temperature of 28 °C. The antifungal activity was assessed by measuring colony diameters to determine the effect of these volatile oils on root rot-associated pathogens in *P. tunicoides*. The inhibition rate was calculated as follows: Inhibition rate (%) = [(colony diameter of control − colony diameter of treatment)/colony diameter of control] × 100%. All data were obtained from three replicates.

#### 4.1.5. Determination of Minimum Inhibitory Concentrations (MICs)

The MICs of the volatile oils were determined according to the method described previously [23]. First, *F. oxysporum* was inoculated into potato dextrose broth (PDB) and incubated at 28 °C on a rotary shaker at 150 rpm for 7 days. After incubation, the culture was filtered through four layers of sterile gauze to remove mycelial debris, centrifuged at 4000 rpm for 10 min at 4 °C to pellet the conidia, and resuspended in sterile water to a final concentration of 1.0 × 10^6^ conidia·mL^−1^. Then, volatile oils and hymexazol were separately dissolved in a mixed solvent system containing 2% (*v*/*v*) DMSO and 0.1% (*v*/*v*) Tween 80 (2-DMSO-T, which differed from 1-DMSO-T only in the concentration of DMSO), followed by filtration through a sterile membrane to obtain filtrates. Subsequently, a series of ten concentration gradients was prepared using a twofold serial dilution method, with an initial concentration of 8 mg·mL^−1^ for both volatile oils and hymexazol. The 96-well plates included a blank control (150 μL of 1/4-strength PDB + 50 μL of 2-DMSO-T), a negative control (150 μL of fungal suspension + 50 μL of 2-DMSO-T), and a positive control (200 μL of fungal suspension). Each treatment had eight replicates and was repeated in three independent 96-well plate experiments. The plates were incubated at 28 °C for 36 h. Following that, optical density (OD) values were measured at 595 nm using a microplate reader (Multiskan GO, Thermo Fisher Scientific, Waltham, MA, USA). The concentration at which fungal growth was completely inhibited, defined as an OD value below 0.1, was used to determine the MIC of volatile oils. MIC values are presented as the mean ± standard deviation (SD) from three independent 96-well plate experiments. These data were processed and statistically analyzed using SPSS 19.0 and Microsoft Excel.

### 4.2. Metagenomic Sequencing and Data Analysis of Rhizosphere Soil from P. tunicoides

#### 4.2.1. Plant Sampling and Processing

The *P. tunicoides* material used in the experiment was obtained from mature seeds cultivated from the native area in Jianchuan County, Dali Bai Autonomous Prefecture, Yunnan Province. Seeds with consistent origin and full grains were selected, sterilized, and then inoculated onto Murashige & Skoog (MS) basal medium supplemented with 1 mg/L 6-benzylaminopurine and 0.1 mg/L 2,4-dichlorophenoxyacetic acid for germination induction. After 30 days, the seedlings were transferred to MS basal medium for continued cultivation. One-year-old seedlings of *P*. *tunicoides* with consistent and healthy growth were selected as experimental materials. The seedlings were transplanted into bare soil collected from Jianchuan County as described above. The experimental treatments included a SYR formulation treatment group, in which natural soil was treated with turpentine extract dispersed in the aqueous formulation system, and a CK group, in which natural soil received sterile water only. Before transplantation, the aerial parts of all plants were removed using sterilized scissors. The root systems were surface-sterilized in 3% sodium hypochlorite solution for 10 min, followed by three rinses with sterile water to ensure the removal of surface contaminants. Then, the treated seedlings were centrally transplanted into plastic pots (16 cm × 14 cm), each containing 500 g of soil. Pine turpentine was diluted in water to its MIC and applied using a slow-release setup. All applications were conducted at a single uniform concentration, specifically the MIC of pine turpentine (NEO) against *F. oxysporum* (0.16 mg·mL^−1^). To be specific, 200 μL of the solution was loaded into a pipette tip, which was positioned approximately 3 cm above the planting site of *P. tunicoides* to allow gradual volatilization. The application was repeated every 3 days. The CK group received an equal volume of sterile water. Each treatment included six replicates, with each replicate comprising ten pots of natural soil, resulting in a total of 120 pots across both treatments. All pots were maintained in controlled growth chambers under identical environmental conditions (average day/night temperatures of 32 °C/22 °C, relative humidity of 60–80%, and a 16 h photoperiod). Pots were randomly rearranged every 3 days to reduce potential positional effects (e.g., variation in light and temperature) within the greenhouse. Soil moisture was adjusted every 2 days with sterile water to maintain approximately 55% water content. After six weeks of cultivation, rhizosphere soil samples were collected from both the SYR and CK groups. Each treatment included six replicates, and each replicate consisted of five plants, with a composite sample pooled by collecting the rhizosphere soil from the five individual plants. Roots were gently shaken to remove loosely attached soil, and the tightly adhering soil (approximately 1–2 mm from the root surface) was collected using a sterile brush and transferred into sterile centrifuge tubes. Samples from the two treatments were processed independently for subsequent metagenomic sequencing. After sampling, the remaining plants were inoculated by applying 10 mL of a *F. oxysporum* conidial suspension (1.0 × 10^7^ conidia·mL^−1^) directly onto the soil surface in each pot. Three weeks later, in the context of symptom development, disease severity was assessed and quantified for *P. tunicoides* plants (*n* = 5 plants per replicate for each treatment).

#### 4.2.2. DNA Extraction and Metagenomic Sequencing

Total microbial DNA was extracted from rhizosphere soil samples using the FastDNA SPIN Soil Kit (MP Biomedicals, Santa Ana, CA, USA) as instructed. A total of 12 samples were obtained, comprising six independent biological replicates per treatment. DNA quality and concentration were determined by agarose gel electrophoresis and spectrophotometric analysis. Metagenomic libraries were subsequently constructed and subjected to paired-end sequencing (150 bp) on the Illumina NovaSeq 6000 platform. Sequencing was performed by Majorbio Bio-Pharm Technology Co., Ltd. (Shanghai, China). Raw sequencing reads were quality-controlled using FastQC v0.11.9, followed by trimming and filtering with Fastp v0.23.3 software [24] to remove low-quality reads and adapter sequences, yielding high-quality clean reads. To eliminate host-derived sequences, clean reads were aligned against the reference plant genome using the BWA v0.7.17 software [71], with the elimination of the matching sequences. The remaining reads were then assembled de novo into contigs using MEGAHIT [72]. The raw sequencing data have been deposited in the National Center for Biotechnology Information (NCBI) Sequence Read Archive database. Open reading frames (ORFs) were predicted from the assembled contigs using Prodigal v2.6.3 software [73]. The predicted ORFs with length ≥ 100 bp were retrieved and translated into amino acid sequences. Redundant sequences were removed using the Cluster Database at High Identity with Tolerance tool with a sequence identity threshold of ≥90% and coverage ≥ 90%, resulting in a non-redundant gene catalog for subsequent analyses. For taxonomic annotation, the non-redundant gene set was aligned against the NCBI non-redundant protein database using BLASTP (E-value ≤ 1 × 10^−5^). Simultaneously, the community composition was profiled across multiple taxonomic levels, including species, genus, family, order, class, phylum, kingdom, and domain. We used Reads Per Kilobase of transcript per Million mapped reads to calculate the relative abundance of genes associated with each genus and its corresponding functional pathways. Functional annotation was performed by aligning gene sequences to the Kyoto Encyclopedia of Genes and Genomes (KEGG), followed by annotation using the KEGG Orthology-Based Annotation System. Functional profiles were generated based on the abundance of KEGG Orthology (KO), pathways, enzyme commission numbers, and modules. Microbial α-diversity metrics were calculated using QIIME2 v2024.10 software [74], and inter-group differences were evaluated using the Wilcoxon rank-sum test. Community structure differences were analyzed using NMDS and Principal Coordinates Analysis (PCoA) based on Bray–Curtis distances, with principal component analysis (PCA) used as a complementary ordination method. The significance of differences between treatments was assessed by PERMANOVA with 999 permutations. Differences in taxonomic abundance among groups were analyzed using the nonparametric Kruskal–Wallis rank-sum test (for multiple comparisons) or the Wilcoxon rank-sum test (for pairwise comparisons). Additionally, linear discriminant analysis was applied to estimate the effect size of differentially abundant taxa, and linear discriminant analysis effect size (LEfSe) was employed to identify key microbial biomarkers across treatments. The metagenomic sequencing data associated with this project have been deposited in the NCBI Short Read Archive database (Accession Number: PRJNA1439554). The detailed database and software information used for metagenomic analysis is provided in Table S10.

#### 4.2.3. Co-Occurrence Network Analysis

To investigate the effects of volatile oil treatment on the potential co-occurrence patterns in the rhizosphere soil of *P. tunicoides*, co-occurrence networks for bacterial and fungal communities were constructed based on Spearman’s rank correlation coefficients among core operational taxonomic units (OTUs). Only robust and significant correlations (|r| > 0.7, *p* < 0.01) were retained. Correlation matrices were calculated using the psych package, and p-values were adjusted using the Benjamini–Hochberg procedure to minimize false-positive associations [75]. Network topological properties, including the number of nodes and edges, average degree, average clustering coefficient, average path distance, modularity index, and positive/negative link ratio, were computed using Gephi 0.10.1. Network visualization was also performed in Gephi.

### 4.3. Isolation, Identification, and Antagonistic Activity Assay of Culturable Bacteria from SYR-Treated Rhizosphere Soil

#### 4.3.1. Isolation and Purification of Culturable Bacteria from Rhizosphere Soil

Culturable biocontrol bacteria were isolated from SYR-treated rhizosphere soil samples collected as described in Section 2.2.1. Briefly, 1 g of rhizosphere soil was added to 9 mL of suspension containing 0.1% tetrasodium pyrophosphate and 1 g of crushed stone, and shaken at 200 rpm for 25 min. Serial dilutions ranging from 10^−2^ to 10^−4^ were then prepared and spread onto 9 cm Petri dishes containing 1/10 Tryptic Soy Agar (TSA) medium, with five replicates for each dilution. After colony formation, single colonies or colony margins were picked using sterile inoculation loops and streaked onto fresh Luria–Bertani (LB) agar medium for purification. The LB medium contained 10 g peptone, 5 g yeast extract, 10 g sodium chloride, 15 g agar, and 1000 mL distilled water per liter. The plates were further incubated at 37 °C to promote colony growth.

#### 4.3.2. 16S rRNA Gene Sequencing and Taxonomic Identification

Bacterial genomic DNA was extracted using a Qiagen DNA extraction kit. The 16S rRNA gene was amplified using the primers 27F (5′-AGAGTTTGATCMTGGCTCAG-3′) and 1492R (5′-TACGGYTACCTTGTTACGACTT-3′), and the PCR products were sequenced by Qiagen Biotechnology Co. (Hilden, Germany). The obtained sequences were compared against the 16S ribosomal RNA database for bacteria and archaea in NCBI using BLAST v2.11.0 to determine their phylogenetic affiliations.

#### 4.3.3. Plate Confrontation Assay Against *F. oxysporum*

To evaluate the in vitro antagonistic activity of the biocontrol bacteria against the pathogenic fungus, a 5 mm diameter fungal plug was placed in the center of freshly prepared PDA medium, and the bacterial isolates were inoculated 3 cm away from the center of the fungal colony. Plates inoculated only with the pathogen were used as controls, and each treatment included six replicates. The plates were incubated in the dark at 28 °C for 7 d. The antagonistic activity of the biocontrol bacteria was evaluated by measuring the colony growth diameter of the pathogenic fungus.

## 5. Conclusions

In conclusion, *P. tunicoides* is frequently found growing in association with *P. yunnanensis* forests, where the incidence of root rot is notably low. The present study provides preliminary insights into the functional characteristics associated with this ecological phenomenon. On the one hand, volatile oils extracted from the roots and needles of *P. yunnanensis* exhibit direct antifungal activity against the principal root rot pathogens of *P. tunicoides*. On the other hand, *P. yunnanensis*-derived volatiles or decomposition products of pine litter may be associated with shifts in the rhizosphere microbiome, as indicated by metagenomic sequencing of rhizosphere soils. These microbial changes may be linked to the observed reduction in root rot severity in *P. tunicoides*, although the underlying causal relationships require further validation. Based on these findings, we propose a working hypothesis in which *P. yunnanensis*-derived volatiles may contribute to the observed reduction in root rot severity mainly through direct pathogen inhibition, with a potential microbiome-associated contribution. This hypothesis should be further validated through experiments designed to distinguish the effects of residual volatiles from those of rhizosphere microbial community changes. Overall, this work provides a preliminary theoretical basis and scientific basis for the development of agroforestry-based cultivation systems for *P. tunicoides*.

## Figures and Tables

**Table 1:** Main chemical components of *P. yunnanensis*-derived volatile oils.

No.	Volatile Oil Name	RT	RI	Compound	CAS NO.	Molecular Formula	Pct Total (%)
NEO	*P. yunnanensis* Turpentine Oil	9.34	937	α-Pinene	80-56-8	C10H16	45.50
		17.03	1189	Longifolene	475-20-7	C15H24	28.20
OEO	*P. yunnanensis* Pine Needle Oil	21.54	1350	α-Terpinyl acetate	80-26-2	C12H20O2	75.13
		19.60	1317	β-Terpinyl acetate	10198-23-9	C12H20O2	7.24

Note: RT, retention time; Pct Total (%), relative peak area proportion based on GC peak area normalization. Compounds were putatively annotated based on NIST 20 library matching; therefore, the results represent putative chemical profiles rather than confirmed quantitative characterization.

**Table 2:** Estimated disease severity and treatment effect under CK and SYR treatments.

Indicator	CK	SYR	Effect Size (SYR − CK)	95% CI for Effect Size
Disease index (DI, %)	51.5	35.5	−16.0 percentage points	−28.3 to −3.7

Note: Disease index was estimated using the midpoint values of grouped disease severity classes: grades 1–3 = 2, grades 4–6 = 5, and grades 7–10 = 8.5. DI was calculated as: $\Sigma (n_{i}\times v_{i})/N\times V_{max}$. Effect size was expressed as the absolute difference in DI between SYR and CK. The 95% CI for the DI difference was estimated from the standard error of the midpoint-derived disease scores and should be interpreted as an approximate interval because the original disease grades were grouped. CI, confidence interval.

## Data Availability

The metagenomic sequencing data generated in this study have been deposited in the NCBI BioProject database under accession number PRJNA1439554. Other data are available from the corresponding author upon reasonable request.

## Supplementary Materials

The following supporting information can be downloaded at: https://www.mdpi.com/article/10.3390/plants15142228/s1. Figure S1: Differential abundance analysis of major microbial taxa contributing to functional potential between the CK and SYR groups; Table S1: Experimental reagents; Table S2: Experimental instruments; Table S3: Chemical Composition of *P. yunnanensis* Turpentine Oil; Table S4: Chemical Composition of *P. yunnanensis* Pine Needle Oil; Table S5: Minimum inhibitory concentrations (MIC, mg/mL) of NEO and OEO against four root-rot pathogens of *P. tunicoides*. Values represent three independent replicates and are expressed as mean ± SD; Table S6: Network topology parameters of bacterial and fungal communities in rhizosphere soils under water (CK) and turpentine oil (SYR) treatments; Table S7: Inhibitory rates (%) of NEO, OEO, Hymexazol, and water control (CK) against four root-rot pathogens of *P. tunicoides*. Values represent the mean of three independent replicates; Table S8: Disease severity rating scale used for evaluating plant root rot symptoms; Table S9: Summary of metagenomic sequencing quality and assembly statistics; Table S10: Key databases and software used for metagenomic analysis.
