# Supplementary material for: Pine-Extracted Volatile Oils Suppress Root Rot in Psammosilene tunicoides Through Direct Antifungal Activity and Rhizosphere Microbiome Modulation
Source: Plants (Basel). 2026 Jul 21;15(14):2228. doi: 10.3390/plants15142228 (PMC13417010; doi:10.3390/plants15142228)
Supplement: Supplementary file 1 [file plants-15-02228-s001.zip › plants-4389508-supplementary.pdf]

Supplementary Figure and Table

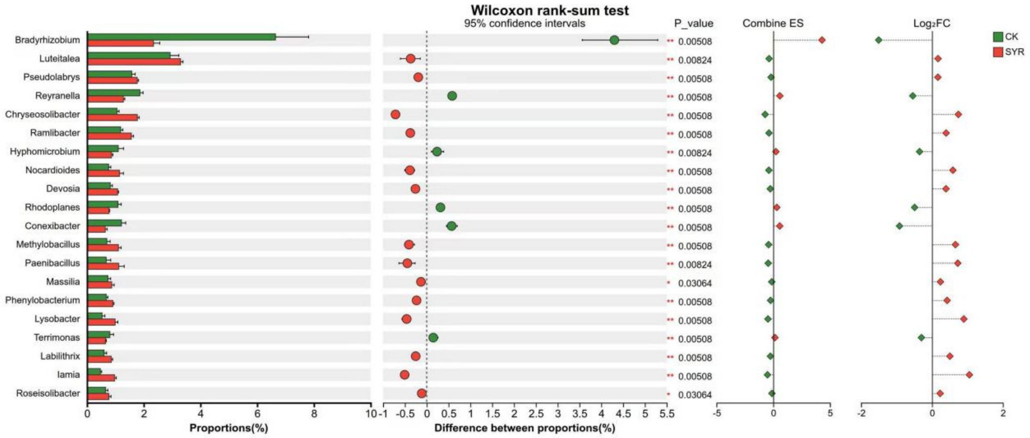

Figure S1. Differential abundance analysis of major microbial taxa contributing to functional potential between the CK and SYR groups.

Table S1. Experimental reagents.

Reagent	Manufacturer	Name
Acetone (AP, 99.5%)	Shanghai Sangon Biological Engineering Technology & Services Co., Ltd.	Acetone (AP, 99.5%)
Cyclohexane (AP, 99.5%)	Tianjin ZhiYuan Reagent Co., Ltd.	Cyclohexane (AP, 99.5%)
Anhydrous sodium sulfate (AP, 99.0%)	Tianjin ZhiYuan Reagent Co., Ltd.	Anhydrous sodium sulfate (AP, 99.0%)
Absolute ethanol (AP, 99.7%)	Tianjin Youpu Reagent Co., Ltd.	Absolute ethanol (AP, 99.7%)
n-Hexane (AP, 98.0%)	Tianjin Fengchuan Chemical Reagent Co., Ltd.	n-Hexane (AP, 98.0%)
DMSO	Shanghai Hengyuan Biotechnology Co., Ltd.	DMSO
Hymexazol	Beijing Zhongke Biochemical Reagent Technology Co., Ltd.	Hymexazol
PDA medium	Guangzhou Ruihua Biotechnology Co., Ltd.	PDA medium

Note: AP, analytical pure; DMSO, dimethyl sulfoxide; PDA, potato dextrose agar.

Table S2. Experimental instruments.

Instrument	Model/Specification	Manufacturer
Ultrapure water system	Likang EASY 30	Nanbei Instrument Limited
Electronic analytical balance	JJ124BC	Shanghai Sangon Biological Engineering Technology & Services Co., Ltd.
Electric heating mantle with temperature control	MH-500/1000	Beijing Kewei Yongxing Instrument Co., Ltd.
Volatile oil extractor	5 mL	Yunnan Feiming Trading Co., Ltd.

Table S2. *Cont.*

Instrument	Model/Specification	Manufacturer
Gas Chromatograph Mass Spectrometer	7890A–5975C	Agilent Technologies, Inc.
Rotary evaporator	OSB-2100	Shanghai Ailang Instrument Co., Ltd.

Table S3. Chemical Composition of *P. yunnanensis* Turpentine Oil

Number	RT	RI	Chemical Composition	CAS NO.	Chemical formula	Pct Total (%)
1	9.338	937	α-Pinene	80-56-8	C10H16	45.50
**2**	**9.787**	**952**	**Camphene**	**79-92-5**	**C10H16**	**1.15**
3	10.615	979	β-Pinene	127-91-3	C10H16	2.63
4	17.034	1189	Longifolene	475-20-7	C15H24	28.20
5	23.634	1419	Caryophyllene	87-44-5	C15H24	8.76
6	21.772	1353	Tricyclo[5.4.0.0(2,8)]undec-9-ene, 2,6,6,9-tetramethyl-, (1R,2S,7R,8R)-	5989-8-2	C15H24	1.29
7	22.393	1374	Longicyclene	1137-12-8	C15H24	1.54
8	22.853	1494	Eremophilene	10219-75-7	C15H24	0.98
9	24.514	1454	Humulene	6753-98-6	C15H24	1.53
10	28.179	1592	Longiborneol	465-24-7	C15H26O	1.54

Note: RT, retention time; RI, retention index; Pct Total (%), relative percentage content.

Table S4. Chemical Composition of *P. yunnanensis* Pine Needle Oil

**Number**	**RT**	**RI**	**Chemical Composition**	**CAS NO.**	**Chemical formula**	**Pct Total(%)**
1	12.116	1031	D-Limonene	5989-27-5	C10H16	0.302
2	17.035	1189	α-Terpineol	98-55-5	C10H18O	0.113
3	17.888	1224	Fenchyl acetate	13851-11-1	C12H20O2	0.388
4	19.597	1317	β-Terpinyl acetate	10198-23-9	C12H20O2	7.241
5	19.704	1350	α-Terpinyl acetate	80-26-2	C12H20O2	3.776
6	19.861	1286	Isobornyl acetate	125-12-2	C12H20O2	0.257
7	20.127	1301	4-Terpinenyl acetate	4821-4-9	C12H20O2	1.239
8	20.604	1315	δ-Terpineol, acetate	93836-50-1	C12H20O2	0.578
9	21.034	1317	β-Terpinyl acetate	10198-23-9	C12H20O2	3.878
10	21.541	1350	α-Terpinyl acetate	80-26-2	C12H20O2	75.128

Table S4. *Cont.*

**Number**	**RT**	**RI**	**Chemical Composition**	**CAS NO.**	**Chemical formula**	**Pct Total(%)**
11	22.813	1365	3-Cyclohexene-1-methanol, 2-hydroxy-α,α,4-trimethyl-	6252-34-2	C10H18O2	1.202
12	23.35	1406	Longifolene	475-20-7	C15H24	1.419

13	23.707	1457	2-((1R,4R)-4-Hydroxy-4-methylcyclohex-2-enyl)propan-2-yl acetate	121958-61-0	C12H20O3	0.327
14	24.047	1149	Ethanone, 1-(1,4-dimethyl-3-cyclohexen-1-yl)-	43219-68-7	C10H16O	0.259
15	24.365	1457	2-((1R,4R)-4-Hydroxy-4-methylcyclohex-2-enyl)propan-2-yl acetate	121958-61-0	C12H20O3	0.286

Note: RT, retention time; RI, retention index; Pct Total (%), relative percentage content.

Table S5. Minimum inhibitory concentrations (MIC, mg/mL) of NEO and OEO against four root-rot pathogens of *P. tunicoides.* Values represent three independent replicates and are expressed as mean ± SD.

Fungus / Essential oil	*Fusarium solani*	*Fusarium oxysporum*	*Rhizoctonia solani*	*Fusarium redolens*
NEO (mg mL^{-1})	0.18 ± 0.11	0.16 ± 0.11	0.50 ± 0.00	0.06 ± 0.03
OEO (mg mL^{-1})	0.44 ± 0.35	0.16 ± 0.13	0.67 ± 0.13	0.65 ± 0.20

Table S6. Network topology parameters of bacterial and fungal communities in rhizosphere soils under water (CK) and turpentine oil (SYR) treatments.

Community	Treatment	Nodes	Edges	Positive correlations	Negative correlations	Average degree	Average path length	Network diameter	Network density	Clustering coefficient	Modularity index
Bacteria	CK	1086	4130	3269	861	7.61	1.13	3.94	0.007	0.986	0.976
	SYR	1000	2841	1746	1095	5.68	1	1	0.0057	1	0.979
Fungi	CK	623	653	382	271	2.10	1	1	0.0034	1	0.991
	SYR	697	787	426	361	2.26	1	1	0.0032	1	0.992

Table S7. Inhibitory rates (%) of NEO, OEO, Hymexazol, and water control (CK) against four root-rot pathogens of *P. tunicoides.* Values represent the mean of three independent replicates.

Pathogen	NEO mean (%)	OEO mean (%)	Hymexazol mean (%)	CK mean (%)
Fusarium solani	87.84	94.71	32.82	6.25
Rhizoctonia solani	91.14	81.65	5.70	3.37
Fusarium oxysporum	93.53	86.72	71.79	2.61
Fusarium redolens	92.57	92.51	72.68	4.33

Table S8. Disease severity rating scale used for evaluating plant root rot symptoms.

Disease grade	Disease description	Scoring criteria
1	Trace disease symptoms	Roots and leaves are largely intact, with no visible black spots, decay, or growth inhibition; only slight root dullness is observed.
2	Very mild disease	A few roots show small black spots or slight decay, with no obvious effect on plant growth.
3	Mild disease	A small area of root decay is observed, some roots are discolored, disease expansion is slow, and plant growth is slightly affected.
4	Mild disease	The roots show obvious decay, some roots are damaged, the disease begins to affect plant growth, and some leaves show slight wilting.
5	Moderate disease	Most roots are decayed, leaves show wilting, some roots are detached, and the disease has clearly expanded.
6	Moderate disease	Root decay is extensive, the plant shows obvious wilting, growth is inhibited, and the disease has spread to multiple roots.
7	Severe disease	Most roots are decayed, the plant is severely wilted, roots can no longer effectively absorb water and nutrients, and leaves become yellow.
8	Severe disease	The roots are almost completely decayed, the root crown/stem base is severely damaged, leaves are dried or have completely lost function, and plant growth has nearly stopped.
9	Severe disease	Extensive root decay and severe root crown/stem base damage are observed; the plant can no longer maintain viability, all leaves are withered, and recovery is difficult.
10	Severe disease	The roots are completely decayed, the plant is dead and cannot recover, and the disease has spread throughout the whole plant.

Table S9. Summary of metagenomic sequencing quality and assembly statistics.

Sample ID	Clean reads	Clean bases (Gb)	Percent in raw reads (%)	Percent in raw bases (%)	Total assembly length (Mb)	Number of contigs	N50 (bp)	N90 (bp)
CK_1	84780646	12.767	98.609	98.340	473.930	983850	533	339
CK_2	80860858	12.177	98.563	98.297	409.574	846029	530	338
CK_3	80337756	12.099	98.721	98.459	456.222	937350	536	340
CK_4	84124464	12.667	98.491	98.212	477.290	964330	542	341
CK_5	84110262	12.665	98.602	98.328	526.748	1059476	543	341
CK_6	83818054	12.621	98.609	98.336	472.497	986429	519	338
SYR_1	78467154	11.817	98.589	98.323	512.229	996459	558	343
SYR_2	80360266	12.101	98.634	98.363	585.704	1110971	578	345
SYR_3	79868888	12.028	98.644	98.381	561.855	1081791	565	343
SYR_4	79721402	12.004	98.671	98.395	604.177	1173630	557	344
SYR_5	79379242	11.953	98.572	98.301	542.643	1041377	564	344
SYR_6	81637346	12.294	98.699	98.429	572.992	1095826	569	344

Table S10. Key databases and software used for metagenomic analysis.

Name	Version	Purpose
NCBI NR	20230830	Taxonomic annotation
KEGG	20230830	Functional annotation
Fastp	v0.20.0	Quality control

MEGAHIT	v1.2.9	Metagenomic assembly
Prodigal	v2.6.3	Gene prediction
CD-HIT	v4.6.1	Non-redundant gene catalog construction
DIAMOND	v2.0.13	Sequence alignment and annotation
SOAPaligner	soap2.21release	Read mapping
Gephi	0.10.1	Co-occurrence network analysis
